# Geographical Variation in Physical Fitness Among Chinese Children and Adolescents From 2005 to 2014

**DOI:** 10.3389/fpubh.2021.694070

**Published:** 2021-09-03

**Authors:** Yanhui Dong, Manman Chen, Yi Song, Jun Ma, Patrick W. C. Lau

**Affiliations:** ^1^Institute of Child and Adolescent Health, School of Public Health, Peking University, Beijing, China; ^2^Department of Sport, Physical Education and Health, Hong Kong Baptist University, Hong Kong, China; ^3^Laboratory of Exercise Science and Health, BNU-HKBU United International College, Zhuhai, China

**Keywords:** physical fitness, geographical variation, children and adolescents, China, trend analysis

## Abstract

**Introduction:** To examine the geographical variation in change in the levels of physical fitness from 2005 to 2014 among Chinese children and adolescents.

**Methods:** A total of 663,813 children 7 to 18 years of age with physical fitness data in 2005, 2010 and 2014, participated in the study. Physical fitness was assessed using six components, and their standardized scores were aggregated to form a summary physical fitness indicator (PFI).

**Results:** Over the study period, there was an increase in the proportion of high PFI (from 15.3% in 2005 to 19.0% in 2014, *P*_trend_ < 0.05) and a decrease in the proportion of low PFI (from 30.6 to 29.8%, *P*_trend_ < 0.05). Children and adolescents in the eastern provinces of China showed a high proportion of high PFI with an increasing trend over time, while those in the western provinces had a high proportion of low PFI with a decreasing trend over time (*P* < 0.05).

**Conclusions:** The comprehensive physical fitness among Chinese children showed an improved trend from 2005 to 2014. Region-specific interventions with priority policies could be useful to sustainably narrow geographical variation in Chinese children, especially in the western provinces.

## Introduction

Physical fitness is the body's ability to perform activities or motions effectively and efficiently. Usually, physical fitness is a comprehensive concept that combines various abilities together, such as muscular strength and endurance, cardiorespiratory endurance, and motor ability (1). Thus, a universal comprehensive physical fitness indicator (PFI) is used to define composite fitness among children and adults (2, 3). Previous studies have physical fitness was associated with the development of cardiovascular diseases (CVD) in both children and adults (4–6).

Physical fitness is considered to be a reflection of outcomes of physical activity (PA), so many physical exercise related factors were considered to affect physical fitness (7–9). Previous study found that the physical fitness among children and adolescents has been a world-wide decline since the late 1950s, especially in the maximal long-distance running performance of Asian children and adolescents, including China (10). One study using a national study in Chinese children and adolescents demonstrated that China has followed global trends in exhibiting a steadily deterioration in the levels of physical fitness, particularly in the domain of cardiorespiratory fitness (11–13).

With the increasing number of factors that contribute to physical fitness deterioration among Chinese children and adolescents, such as the increased prevalence in obesity, lack of physical exercise, increased academic burden and use of electronic devices, more public health measures need to be focused on priority areas and regions. However, there has been a lack of systematic research in examining changes in geographic variations of comprehensive physical fitness using population-level prevalence data. Therefore, the objective of this study is to examine geographical variation in the levels of physical fitness among Chinese school-aged children and adolescents using three successive national cross-sectional surveys from 2005 to 2014.

## Method

### Study Design and Participants

Data were extracted from CNSSCH in 2005, 2010 and 2014. The CNSSCH was the largest nationally representative surveys comprising multiple core components of physical fitness among Chinese children and adolescents aged 7–18 years, and the CNSSCH was jointly launched by the Ministry of Education, the Ministry of Health, the Ministry of Science and Technology, the State of Nation Affairs, and the State Sports General Administration of People's Republic of China in order to investigate the constitution and health of children and adolescents. The details of the design of CNSSCH have been described in the previous studies (14). The CNSSCH used a multistage stratified cluster sampling design and included 31 Chinese provinces but did not cover Hong Kong, Macau and Taiwan. Initially, children and adolescents in each province, except Tibet, were stratified by three levels of prefecture cities (i.e., upper, moderate, low) based on their socioeconomic status. Then, in each city of three sub-provincial levels, children and adolescents were also stratified by urban and rural areas based on their residence location. Within these stratified areas since 1985, schools covering students aged 7–18 years including primary, middle and high schools were randomly selected, and the sampling procedure has remained uniform in each subsequent survey year. From these schools, all students in the randomly selected classes were included in the survey. Following inclusion and exclusion criteria and obtaining informed consent (as shown in previous studies) (14, 15), at least 50 Han students in each age group from 7–18 years of boys or girls in urban and rural areas were included in the survey. As participants in Tibet were minority students, only children in Lasa city were surveyed for feasibility reasons. At last, a total of 663,813 children and adolescents aged 7–18 years (234,289 in 2005, 215,223 in 2010, and 214,301 in 2014) were collected for the geographical variations trends analyses of physical fitness.

### Physical Fitness Measurements and Categories

Participants in the survey underwent a complete physical fitness test according to the same protocol at all survey sites. All physical fitness tests were implemented in PE classes by special trained PE teachers who have passed the measurement examination. A school doctor was present to prevent children and adolescents from being injured during the physical fitness test, and a project supervisor was present to monitor whether the physical fitness test was carried out as required and to provide necessary guidance. Six physical fitness components (forced vital capacity (FVC), standing long jump (SLJ), sit-and-reach (SR), body muscle strength (BMS), 50 meter dash and endurance running) were included in our study and were measured by a team of trained technicians following a standardized procedure. FVC, SLJ, SR, and 50 meter dash were measured at each age from 7–18 years. According to the changing physical capabilities with age and sex, BMS was evaluated by oblique body pull-ups (boys aged 7–12 years), pull-ups (boys aged 13–18 years) and 1 min sit-ups (girls aged 7–18 years). Speed and speed endurance were evaluated by interval 50 m dash time and time for long distance running (50 m × 8 shuttle run for both boys and girls aged 7–12 years; 1,000 m endurance running for boys aged 13–18 years; and 800 m endurance running for girls aged 13–18 years). All the measuring instruments were consistent at each survey site and calibrated before use. All the students in the final analysis took each test simultaneously. Almost 100% of the students took all tests on the same day. The sample sizes and proportion of participants in each item test in each survey year are shown in the Supplementary Table 1. A total of 234,289, 215,223, and 214,301 chidlren and adolescents participated the physical fitness tests, and total valid participants for each physical fitness component tests in 2005 and 2010 exceeded 99.0% and even reached at 100% in 2014.

Allowing for the different items in boys and girls of different age groups and difficulties of comparison, sex- and age-specific standardized values were calculated for each physical fitness component. Based on the reference population with median values and standard deviation (SD) of each physical fitness component that was defined in previous studies (2, 12). Z scores of each component were calculated as an individuals' item value minus the median, divided by the SD for that child's age and sex in the reference population. PFI was calculated by summing the standard values for each of the six items: *PFI* = *standardized values of FVC* + *SLJ* + *SR* + *BMS* +*(*−*50 m dash)*+*(–endurance running)*. Based on the percentile of the PFI in the reference population in 1985, we categorized the PFI into five levels: low level (<20th), low-middle level (≥20th and <40th), middle level (≥40th and <60th), middle-high level (≥60th and <80th), high level (≥80th).

### Statistical Analysis

Descriptive statistics were used to describe the demographic characteristics of the study population in CNSSCH. Chi-square tests were used to examine between group differences in categorical variables. Stacking scales diagrams were employed to examine trends in the levels of physical fitness across the study period. To analyze geographical variation, we compared the proportion of different levels of physical fitness in each province (excluding Hong Kong, Macau, and Taiwan) across the three survey years. To examine geographical variation, the thirty-one mainland Chinese provinces were divided into four regions: East, Central, West, and Northeast, in accordance with the geographical standard division from the Chinese National Bureau of Statistics (NBS) (Supplementary Figure 1) (16). We used the Cochran-Armitage trend test to calculate the *P*_trend_ values. Statistical analyses were conducted with Stata (version 15.0) statistical software. Two-sided *P* < 0.05 was considered statistically significant.

## Results

### Study Population Characteristics

The characteristics of the study population of CNSSCH are presented in Supplementary Table 1. The proportion of sample sizes for physical fitness from 2005 to 2014 and questionnaire survey in 2014 were balanced across sex, age, provinces, and urban and rural areas. The mean of original and standardized values of FVC got improvement, but other five physical fitness components among children and adolescents presented a fluctuating tends from 2005 to 2014 (Table 1).

**Table 1:** The sample size and distribution of each physical fitness component from 2005 to 2014 in CNSSCH.

**Survey year**	**2005**	**2010**	**2014**
Total sample size, n	234,289	215,223	214,301
**Physical fitness measurement**			
Forced vital capacity, *n* (%)	234,003 (99.88)	215,171 (99.98)	214,301 (100.00)
Original values (Mean (SD), ml)	2,017.1 (923.0)	2,095.4 (947.1)	2,210.0 (1,001.8)
Standardized values, Mean (SD)	−0.88 (1.27)	−0.66 (1.33)	−0.41 (1.41)
Standing Long Jump, *n* (%)	234,229 (99.97)	215,001 (99.90)	214,301 (100.00)
Original values (Mean (SD), cm)	165.9 (37.0)	166.4 (37.5)	163.5 (38.1)
Standardized values, Mean (SD)	0.25 (1.09)	0.28 (1.13)	0.12 (1.17)
Sit and Reach, *n* (%)	234,241 (99.98)	214,983 (99.89)	214,301 (100.00)
Original values (Mean (SD), cm)	9.2 (6.8)	9.4 (6.7)	9.1 (7.1)
Standardized values, Mean (SD)	0.14 (1.19)	0.19 (1.17)	0.14 (1.23)
Body Muscle Strength, *n* (%)	234,086 (99.91)	214,741 (99.78)	214,301 (100.00)
Original values, Mean (SD):			
Oblique Body Pull-ups (Boys aged 7–12 years)	30.8 (22.6)	26.2 (18.4)	23.8 (16.0)
Pull-Ups (Boys aged 13–18 years)	3.9 (4.1)	4.0 (5.1)	3.4 (3.6)
1-min Sit-Ups (Girls aged 7–18 years)	26.3 (10.8)	25.3 (10.8)	27.8 (10.8)
Standardized values, Mean (SD)	0.39 (1.33)	0.27 (1.24)	0.32 (1.22)
50 Meter Dash, *n* (%)	234,164 (99.95)	214,884 (99.84)	214,301 (100.00)
Original values (Mean (SD), s)	9.5 (1.5)	9.6 (1.5)	9.5 (1.5)
Standardized values, Mean (SD)	−0.02 (1.24)	0.01 (1.26)	−0.07 (1.28)
Endurance running, *n* (%)	233,301 (99.58)	213,719 (99.30)	214,301 (100.00)
Original values (Mean (SD), s)			
50 m ×8 shuttle run (Students aged 7–12 years)	126.7 (19.3)	127.1 (17.3)	126.7 (17.3)
1,000 m run (Boys aged 13–18 years)	272.1 (38.3)	271.5 (39.0)	274.3 (41.2)
800 m run (Girls aged 13–18 years)	261.7 (32.4)	260.9 (32.8)	261.5 (34.2)
Standardized values, Mean (SD)	1.00 (1.52)	1.03 (1.56)	1.02 (1.55)

*n (%), the sample size and percentage completing each physical fitness measurement*.

### Trend in Physical Fitness

**Figure 1:**
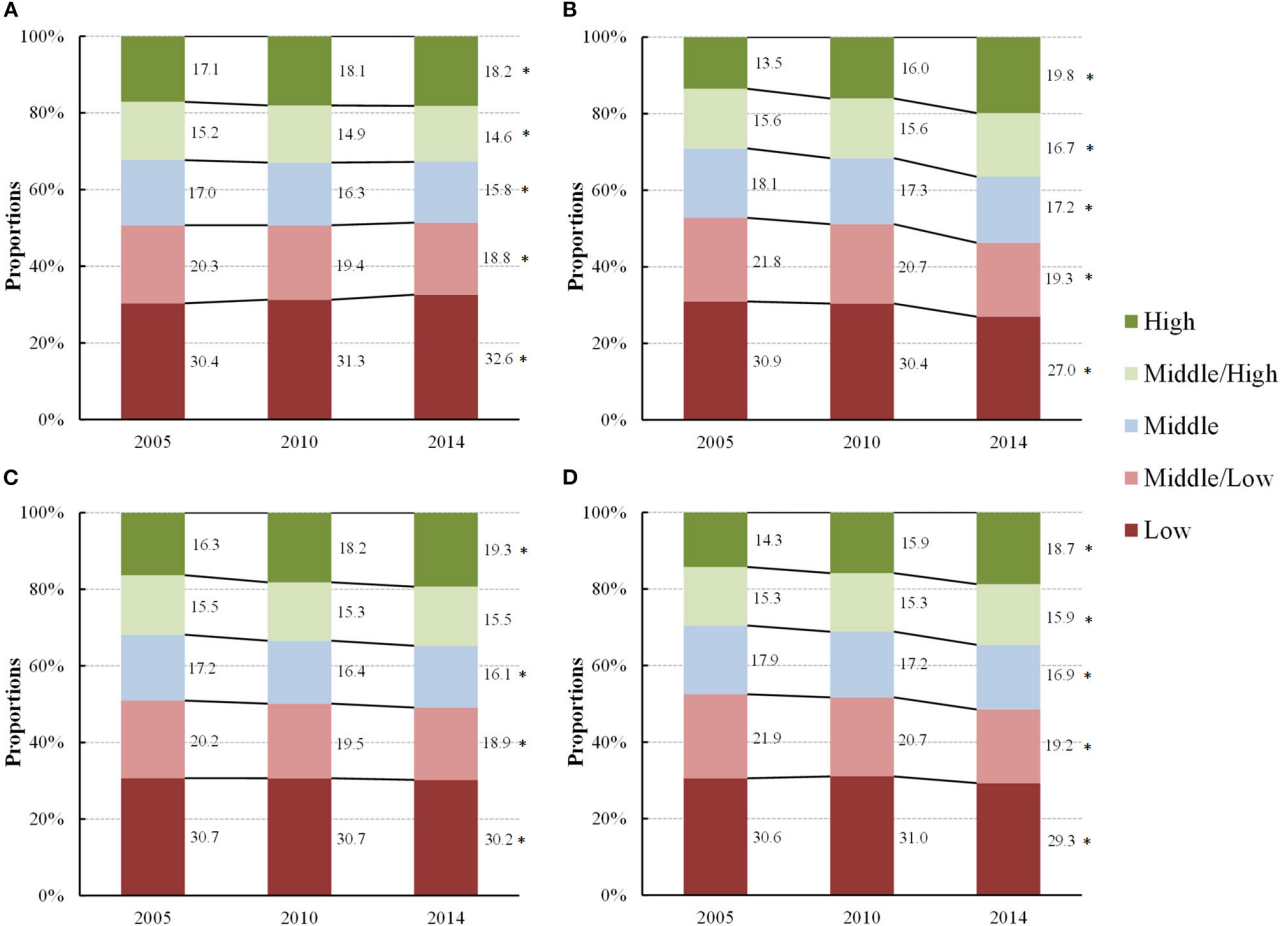
Proportions of change in different levels of physical fitness, as indexed by physical fitness index, in children and adolescents by sex and residence [**(A)** Boys, **(B)** Girls, **(C)** Urban and **(D)** Rural] from 2005 to 2014 (2005–2014, China). ^*^A statistically significant increased trend or decreased trend in the proportions of each level of physical fitness from 2005 to 2014 (*P*_trend_ < 0.05) using Cochran-Armitage trend test.

**Figure 2:**
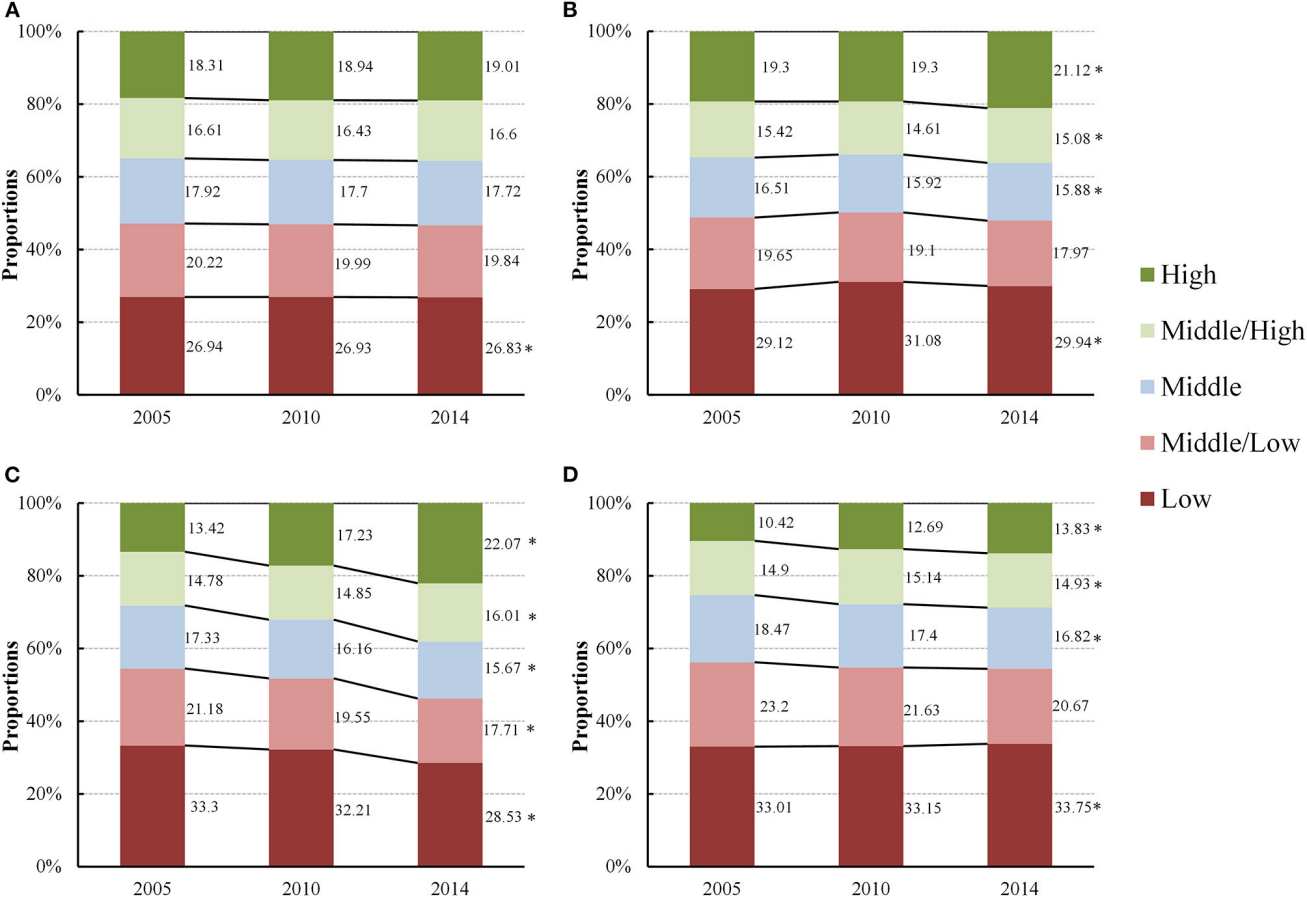
Proportions of change in different levels of physical fitness, as indexed by physical fitness index, in children and adolescents by age groups [**(A)** 7–9 years, **(B)** 10–12 years, **(C)** 13–15 years and **(D)** 16–18 years] from 2005 to 2014 (2005–2014, China). ^*^A statistically significant increased trend or decreased trend in the proportions of each level of physical fitness from 2005 to 2014 (*P*_trend_ < 0.05) using Cochran-Armitage trend test.

Physical fitness, as indexed by PFI, improved with an increased proportion of children and adolescents who performed at above middle-high levels of PFI (*P*_trend_ < 0.05), and a decreased proportion of those who scored below low-middle levels of PFI (*P*_trend_ < 0.05). Boys and girls showed a similar increasing trend in the proportion of their physical fitness from 2005 to 2014 with both girls and boys showing a significant increase in the proportion of high levels of PFI (13.5% in 2005 to 19.8% in 2014, *P*_trend_ < 0.05) for girls, 17.1% in 2005 to 18.2% in 2014, *P*_trend_ < 0.05 for boys, respectively). The proportion of low levels of PFI decreased in girls, but increased in boys (30.9–27.0% in girls, *P*_trend_ < 0.05, and 30.4–32.6% in boys, *P*_trend_ < 0.05, respectively). With respect to residence, results from urban and rural students revealed a similar trend, showing an increased proportion of those who performed above middle-high levels of PFI and a decreased proportion of those who performed below low-middle levels of PFI (*P*_trend_ < 0.05) (Figure 1). Among the four age groups, the proportion of the high levels of PFI showed an increased trend from 2005 to 2014, particular for those aged 13–15 years from 13.4 to 22.1% during the 10 years (*P*_trend_ < 0.05). Also, children and adolescents aged 13–15 years witnessed the largest decrease for low levels of PFI from 33.3% in 2005 to 28.5% in 2014 (*P*_trend_ < 0.05) (Figure 2).

### Geographical variations

**Figure 3:**
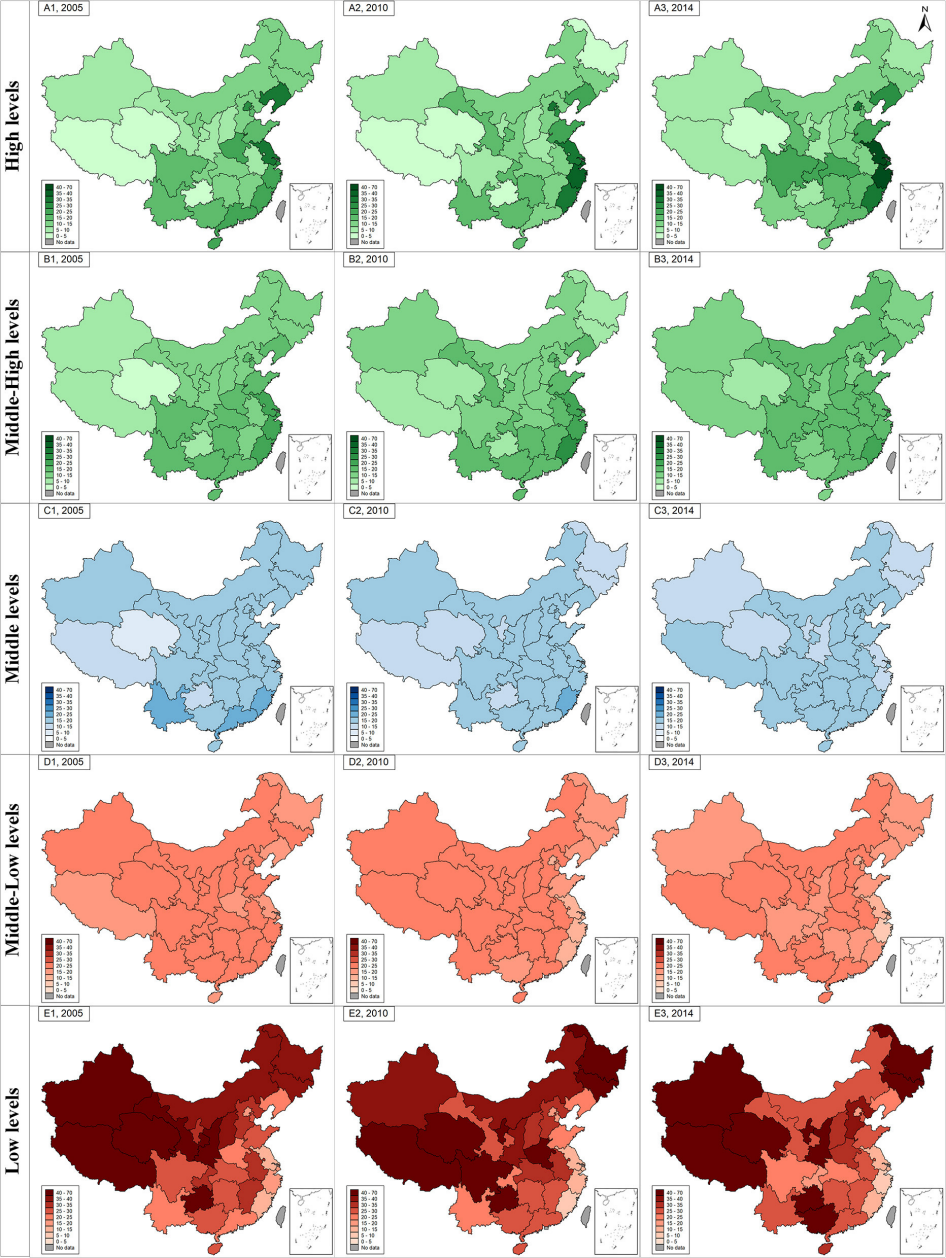
The geographical distribution and changes of different levels of physical fitness index from 2005 to 2014 (2005–2014, China). The green graph in subfigure (**A1**, **A2**, and **A3** for 2005, 2010, and 2014) and (**B1**, **B2**, and **B3** for 2005, 2010, and 2014) represents geographical distribution of high levels of PFI and middle-high levels of PFI in 2005, 2010, and 2014. The blue graph in subfigure (**C1**, **C2**, and **C3** for 2005, 2010, and 2014) represents geographical distribution of middle levels of PFI in 2005, 2010, and 2014. The red graph in subfigure (**D1**, **D2**, and **D3** for 2005, 2010, and 2014) and (**E1**, **E2**, and **E3** for 2005, 2010, and 2014) represents geographical distribution of low levels of PFI and middle-low levels of PFI in 2005, 2010, and 2014. The gradient of each color series in each subfigure was used to show the exact proportions of the specific PFI levels (exact number showed in Supplementary Table 2).

With regard to the level of PFI (data shown in Figure 3), over the study period, children living in eastern Chinese provinces showed a relatively higher proportion of physical fitness compared to those living in western provinces. For example, children in the eastern coastal provinces, such as Jiangsu, Zhejiang, Shanghai, Beijing, and Fujian, exhibited the largest proportion of high level of PFI. Up to 2014, the proportions of high level of PFI in the eastern, central, western and northeastern regions were 28.5, 16.4, 12.5, and 16.5%, respectively (Table 2). Students living in the eastern regions also witnessed a largest increase in the proportion of high level of PFI (6.7 percent point from 2005 to 2014), followed by those living in the western and central regions (2.8 and 2.0 percent point from 2005 to 2014), but those from northeastern regions even showed a decrease in high level of PFI from 17.8% in 2005 to 16.5% in 2014 with 1.3 percent point decreases.

**Table 2:** The geographical changes of different levels of physical fitness index from 2005 to 2014.

**Categories**	**2005, %**	**2010, %**	**2014, %**	2005-2014 Changes (Percent point, *P* values) ^*^
High Level				
East	21.8	27.0	28.5	6.7 (<0.001)
Central	13.4	12.0	16.4	3.0 (<0.001)
West	9.6	11.7	12.5	2.8 (<0.001)
Northeast	17.8	13.7	16.5	−1.3 (0.001)
Middle-High level				
East	18.7	19.3	17.6	−1.2 (<0.001)
Central	15.5	14.4	16.4	0.9 (0.001)
West	12.7	13.1	14.0	1.3 (<0.001)
Northeast	14.2	11.7	14.0	−0.1 (0.739)
Middle Level				
East	18.9	17.7	16.0	−3.0 (<0.001)
Central	18.2	18.1	18.1	−0.1 (0.701)
West	16.6	16.2	16.7	0.1 (0.634)
Northeast	15.5	13.5	14.8	−0.7 (0.053)
Low-Middle Level				
East	19.6	17.2	16.3	−3.3 (<0.001)
Central	22.2	22.8	20.6	−1.6 (<0.001)
West	22.4	21.7	20.9	−1.5 (<0.001)
Northeast	19.4	18.0	18.2	−1.2 (0.002)
Low Level				
East	21.0	18.8	21.6	0.7 (0.002)
Central	30.8	32.8	28.6	−2.2 (<0.001)
West	38.7	37.4	36.0	−2.7 (<0.001)
Northeast	33.2	43.1	36.4	3.2 (<0.001)

* *The difference of proportions of each level of PFI in different survey years between 2005 and 2014 years was evaluated by the Chi-square test*.

**Figure 4:**
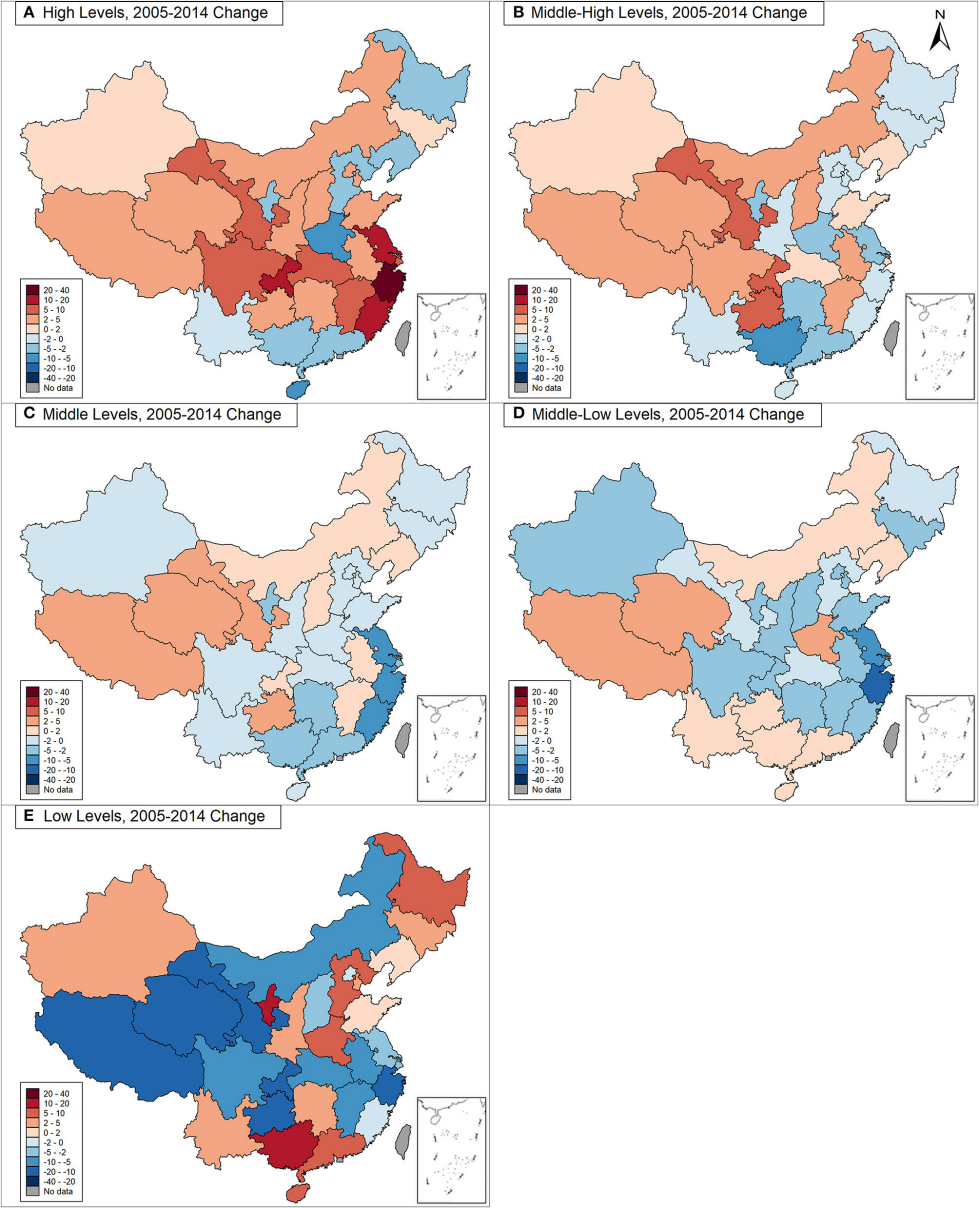
The changes of different levels of physical fitness from 2005 to 2014 (2005–2014, China). **(A)** For high levels of PFI; **(B)** for middle-high levels of PFI; **(C)** for middle levels of PFI; **(D)** for middle-low levels PFI; **(E)** for low levels PFI. The red-colored item in subfigure represents an increased percentage point of each level physical fitness index from 2005 to 2014. The blue-colored item in subfigure represents a decreased percentage point of each level physical fitness index from 2005 to 2014. The color gradient of red and blue series was used to show the proportions' changes of each level of PFI (exact number showed in Supplementary Table 2).

On the contrary, children and adolescents in the western and northeastern provinces showed a high proportion of low levels of PFI, followed those living in the central and eastern provinces. The proportions of low levels of PFI in the eastern, central, western and northeastern provinces in 2005 were 21.0, 30.8, 38.7, and 33.2% respectively. In 2014, their estimates were changed to 21.6, 28.6, 36.0, and 36.4%, respectively. Across the four geographic regions, children and adolescents in the western provinces showed the largest decrease (by 2.7 percentage points) in the proportion of low levels of PFI from 2005 to 2014, though there was also an increase, between 2005 and 2014, for those living in the eastern and northeastern provinces. Specifically, children and adolescents in Gansu, Qinghai, and Tibet were shown to have the largest decrease in the proportion of low levels of PFI (by 15.7, 15.4, and 15.1% points). In contrast, children and adolescents in Guangxi and Ningxia exhibited the largest increase in the proportion of low levels of PFI (by 15.0 and 14.7% points, respectively (Figure 4 and Supplementary Table 2). The standardized values of each physical fitness components were also showed in the Supplementary Table 3. The FVC, 50 meter dash, and endurance running among children and adolescents got improvement from 2005 to 2014 in four regions, particularly in central and western regions, but the standing long jump, sit and reach and body muscle strength deteriorated in 2014 than they were in 2005.

## Discussion

To our knowledge, this is the first study that used three successive national cross-sectional surveys between 2005 and 2014 that assessed the geographic variations in comprehensive physical fitness among Chinese school-aged children and adolescents. From 2005 to 2014, there was an increase in the proportion of high PFI and a decrease in the proportion of low PFI among Chinese children and adolescents, especially among girls, and those living in rural areas and in middle-school stage. These trends were observed with notable geographic variability showing that children and adolescents in the eastern provinces having a higher proportion of PFI compared with a higher proportion of low PFI in children and adolescents living in the western provinces. During the study period, the levels of physical fitness among children and adolescents in both eastern and western provinces showed an upward (in the direction of improvement) trend.

Actually, there has been an increase in health-promotion policies at national and local levels. In 2007, the State Council of the Chinese government issued a strategic policy aimed at strengthening and enhancing physical fitness for school-aged children and adolescents (17). In the years followed, the government issued a total of 88 multisectoral measures and policies involving promotion of sport, reductions in academic burden through curriculum reform, and development of public facilities to promote students' physical fitness (12, 18). Although there has been an increase in health-promotion policies in China, our findings highlight notable geographic variations in the levels of children's physical fitness across regions with children living in the eastern provinces experiencing high levels of fitness compared to those living in the western provinces. These geographic disparities may reflect an uneven development and implementation of nationwide policies. In order to promote physical fitness among children and adolescents, China initiated its Healthy China 2030 blueprint in 2016 (19). It set out specific requirements on physical activities for children and puts them into action, which makes school-aged children achieve at least 1 h of physical activity daily and have more than 25% of them achieve an “excellent” rating in physical fitness. Thus, the geographic variations in physical fitness in this study will clarify the disadvantaged areas and the priority of public health resources investment under the national Healthy China 2030 strategy.

Findings from this study highlight significant geographic variations in the levels of fitness among children and adolescents living between economically developed regions (e.g., eastern provinces) and those that are less well-developed (e.g., western provinces). It is conceivable that such variation may reflect geographic disparities in economic development, investment in physical activity or sport facilities, and inherent uneven resources. As such, in order to narrow these disparities, targeted physical fitness interventions or programs should be directed toward school children living in those under developed areas (e.g., western provinces). From the health promotion perspective, priority areas in physical fitness programs need to be identified across regions of a wide socioeconomic spectrum and be targeted at the province, district, school and classroom levels, through policy and practice recommendations, periodical physical fitness and health surveillance, and implementation of evidence-based health-related school PE curricula (20–22).

Apart from those nationwide efforts, reductions in academic pressures, breaking of sedentary time, and engagement in sporting activities should also be encouraged to narrow these regional disparities in children and adolescents' physical fitness (23). For example, since obesity and undernutrition were both important causes of physical fitness deterioration, the background of rapid increase in obesity and persistent undernutrition among children and adolescents suggested that the differentiated improvement measures were needed in different regions of China (24, 25). Thus, as suggested in the previous study, the control of over-nutrition should be emphasized among eastern developed provinces with policies that encourage taxation on unhealthy foods and promotion of moderate-to-vigorous PA. In the less developed western provinces, emphasis should be placed on improving malnutrition with promotion of rural nutrition improvement programs and dietary diversity (12).

Our study has several strengths, derived from its comprehensive assessment of physical fitness combining respiratory function, strength, flexibility, explosive power, and cardiorespiratory endurance factions, with a large nationally-representative sample size and repeated measures with geographic variations across a 10-year period. Our study also has several limitations that should be noted. First, since this study used original data on subnational changes from three cross-sectional surveys, the data cannot be used to infer causality, and merely ecological analyses were adopted in this study. However, the CNSSCH collected nationally representative data with a large sample size and compelling evidence, and the comprehensive physical fitness estimated in CNSSCH could meet our purpose of secular trend, geographical and influencing factors analyses. Second, our study adopted the PFI assessing the comprehensive physical fitness, and did not use the national fitness standard, and also some characteristics of six components of PFI might be ignored. The scope of PFI could be not as broad as the national fitness standard in the use of the individual evaluation of physical fitness. But it has other advantages, for example, it was helpful to observe the different levels of physical fitness, including the low, low-middle, middle, middle-high, and high levels of PFI analyzed in this study, and extend the application scope in the use of PFI in terms of trend analysis and intra-data comparison. In addition, the national fitness standards were calculated based on grade rather than age, which did not apply to our data.

## Conclusions

Findings from this study showed an improved trend, from 2005 to 2014, in the overall level of physical fitness among Chinese children and adolescents. However, notable geographic variations in the levels of fitness were observed with high prevalence of healthy fit children and adolescents living in the eastern region and unhealthy or low-fit children and adolescents living in the western region of the country. These findings suggest that national and regional school fitness health-enhancing strategies and initiatives should aim to reduce geographic disparities by targeting evidence-based interventions in geographical regions that have a high prevalence of unhealthy physical fitness in school children and adolescents across the country from diverse regions.

## Data Availability Statement

All the individual (de-identified) participant data collected in the surveys can be shared with investigators whose proposed use of the data has been approved by an independent review committee identified for this purpose by contacting the corresponding author. Proposals should be directed to majunt@bjmu.edu.cn and songyi@bjmu.edu.cn.

## Ethics Statement

The studies involving human participants were reviewed and approved by the Medical Research Ethics Committee of the Peking University Health Science Center. Written informed consent to participate in this study was provided by the participants' legal guardian/next of kin.

## Author Contributions

YD conceptualized and designed the study, completed the statistical analyses, drafted the initial manuscript, and reviewed and revised the manuscript. JM and YS contributed to the conceptualization and design of the study, supervised the data collection, the statistical analyses and initial drafting of the manuscript, and reviewed and revised the manuscript. PL participated in conceiving the study design and critically reviewed and revised the manuscript from preliminary draft to submission. MC assisted with the data interpretation, and reviewed and revised the manuscript. All authors approved the final manuscript as submitted and agreed to be accountable for all aspects of the work.

## Conflict of Interest

The authors declare that the research was conducted in the absence of any commercial or financial relationships that could be construed as a potential conflict of interest.

## Publisher's Note

All claims expressed in this article are solely those of the authors and do not necessarily represent those of their affiliated organizations, or those of the publisher, the editors and the reviewers. Any product that may be evaluated in this article, or claim that may be made by its manufacturer, is not guaranteed or endorsed by the publisher.
