# Supplementary material for: Geographical Variation in Physical Fitness Among Chinese Children and Adolescents From 2005 to 2014
Source: Front Public Health. 2021 Sep 3;9:694070. doi: 10.3389/fpubh.2021.694070 (PMC8446346; doi:10.3389/fpubh.2021.694070)
Supplement: Supplementary file 1 [file Data_Sheet_1.DOCX]

Supplementary Material


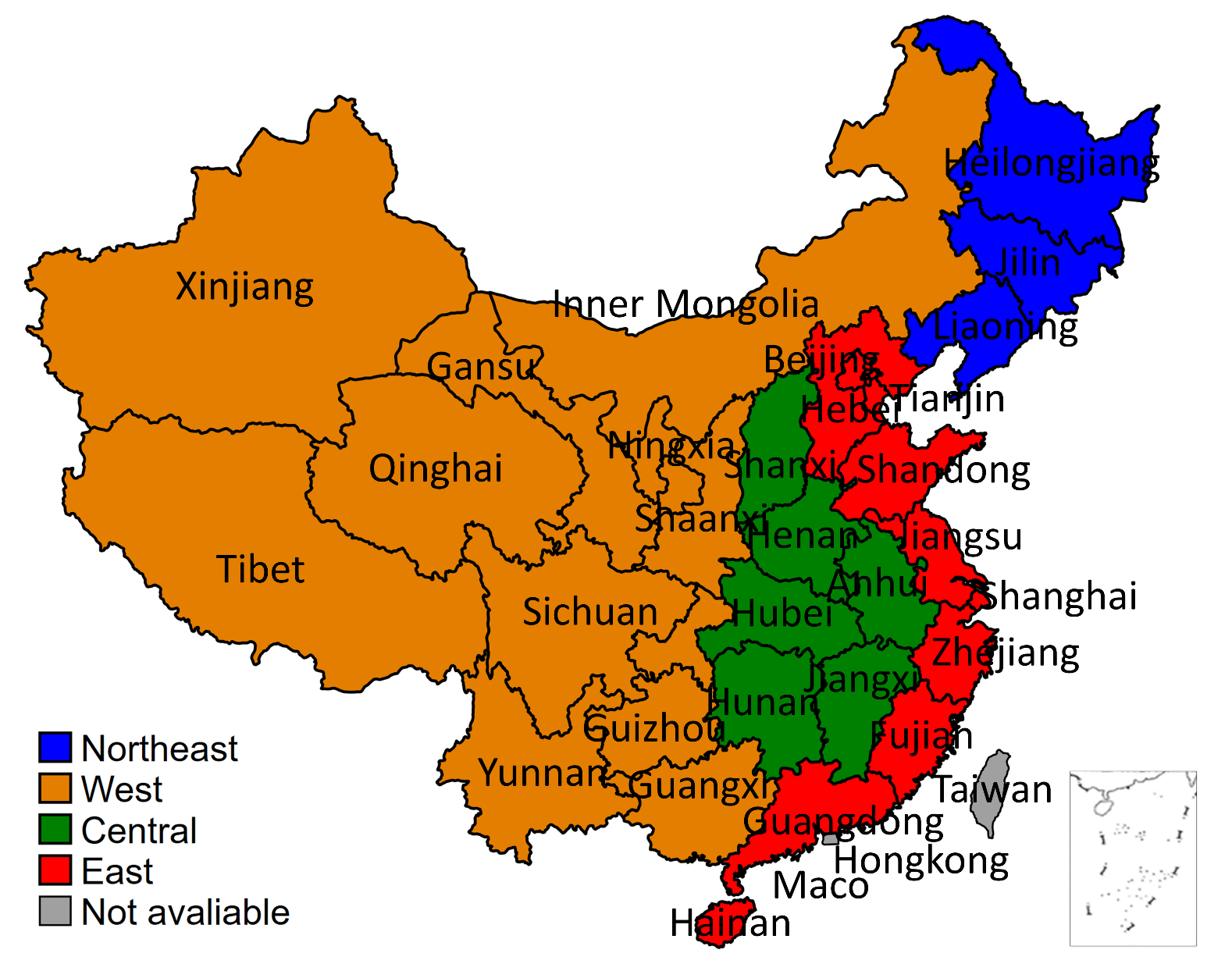


**Figure S1**. The geographical classification in each province for east, central, west and northeast regions in China

Note: Three colors presented east (red), central (green), west (orange) and northeast (blue) regions of China. The CNSSCH included the 31 mainland provinces and excluded Hong Kong, Macao, and Taiwan.

| **Table S1.** Basic information of physical fitness surveys from 2005 to 2014 among Chinese children and adolescents (2005 to 2014, China) | | | |
| --- | --- | --- | --- |
| Variables | 2005 | 2010 | 2014 |
| Sample | 234289 | 215223 | 214301 |
| **Gender**, n(%) |  |  |  |
| Boys | 117594(50.2) | 107611(50.0) | 107175(50.0) |
| Girls | 116695(49.8) | 107612(50.0) | 107126(50.0) |
| **Areas**, n(%) |  |  |  |
| Urban | 117932(50.3) | 107537(50.0) | 107211(50.0) |
| Rural | 116357(49.7) | 107686(50.0) | 107090(50.0) |
| **Regions**, n(%) |  |  |  |
| East | 79790(34.1) | 71644(33.3) | 71384(33.3) |
| Central | 42553(18.2) | 43124(20.0) | 43047(12.1) |
| West | 88944(38.0) | 78917(36.7) | 78381(11.7) |
| Northeast | 23002(9.8) | 21538(10.0) | 21489(10.0) |
| **Age groups**, n(%) |  |  |  |
| 7-9y | 58041(24.8) | 53801(25.0) | 53665(25.0) |
| 10-12y | 58441(24.9) | 53856(25.0) | 53759(25.1) |
| 13-15y | 58448(24.9) | 53857(25.0) | 53858(25.1) |
| 16-18y | 59359(25.3) | 53709(25.0) | 53019(24.7) |

Note: n(%) represents the sample and its percentage.

| **Table S2**. The geographical distribution and changes of different levels of physical fitness from 2005 to 2014 | | | | | | | |
| --- | --- | --- | --- | --- | --- | --- | --- |
| Regions | Low level PFI | | |  | High level PFI | | |
|  | 2005 | 2014 | 2005-2014 |  | 2005 | 2014 | 2005-2014 |
| Eastern provinces | 21.0 | 21.6 | 0.7 |  | 21.8 | 28.5 | 6.7 |
| Beijing | 17.1 | 16.4 | -0.7 |  | 28.8 | 32.9 | 4.1 |
| Tianjin | 25.9 | 28.4 | 2.5 |  | 16.6 | 19.9 | 3.3 |
| Hebei | 32.0 | 38.3 | 6.3 |  | 14.3 | 12.1 | -2.2 |
| Shanghai | 16.0 | 13.3 | -2.8 |  | 25.5 | 35.4 | 9.9 |
| Jiangsu | 13.3 | 11.0 | -2.3 |  | 30.6 | 45.8 | 15.2 |
| Zhejiang | 18.1 | 5.6 | -12.5 |  | 21.7 | 57.1 | 35.4 |
| Fujian | 14.0 | 13.1 | -1.0 |  | 20.5 | 32.1 | 11.5 |
| Shandong | 26.8 | 26.9 | 0.0 |  | 18.0 | 21.2 | 3.2 |
| Guangdong | 20.2 | 29.2 | 9.1 |  | 20.0 | 15.7 | -4.3 |
| Hainan | 25.8 | 33.7 | 7.9 |  | 20.9 | 13.9 | -7.0 |
| Central provinces | 30.8 | 28.6 | -2.2 |  | 13.4 | 16.4 | 3.0 |
| Shanxi | 37.8 | 33.4 | -4.4 |  | 9.6 | 13.9 | 4.3 |
| Anhui | 34.1 | 27.3 | -6.8 |  | 9.9 | 14.0 | 4.2 |
| Jiangxi | 34.0 | 28.3 | -5.7 |  | 11.5 | 17.3 | 5.9 |
| Henan | 25.0 | 31.9 | 7.0 |  | 20.3 | 13.2 | -7.1 |
| Hubei | 27.8 | 22.0 | -5.8 |  | 12.7 | 20.2 | 7.5 |
| Hunan | 26.3 | 28.5 | 2.2 |  | 14.9 | 19.7 | 4.9 |
| Western provinces | 38.7 | 36.0 | -2.7 |  | 9.6 | 12.5 | 2.8 |
| Chongqing | 32.3 | 18.9 | -13.4 |  | 10.6 | 22.4 | 11.8 |
| Sichuan | 29.6 | 24.1 | -5.6 |  | 15.5 | 23.9 | 8.5 |
| Guizhou | 55.4 | 41.5 | -13.9 |  | 2.9 | 6.3 | 3.5 |
| Yunnan | 22.8 | 26.9 | 4.2 |  | 15.8 | 14.3 | -1.5 |
| Shaanxi | 43.5 | 47.0 | 3.5 |  | 7.1 | 9.5 | 2.3 |
| Inner Mongolia | 39.7 | 29.8 | -9.9 |  | 10.3 | 14.4 | 4.1 |
| Gansu | 41.2 | 25.6 | -15.7 |  | 7.3 | 15.6 | 8.4 |
| Qinghai | 63.1 | 47.7 | -15.4 |  | 2.2 | 4.8 | 2.6 |
| Ningxia | 32.6 | 47.3 | 14.7 |  | 11.3 | 6.5 | -4.8 |
| Xinjiang | 45.7 | 48.3 | 2.6 |  | 6.1 | 7.8 | 1.6 |
| Guangxi | 26.1 | 41.1 | 15.0 |  | 15.5 | 10.5 | -4.9 |
| Tibet | 60.1 | 45.0 | -15.1 |  | 4.2 | 6.8 | 2.6 |
| Northeast provinces | 33.2 | 36.4 | 3.2 |  | 17.8 | 16.5 | -1.3 |
| Liaoning | 20.3 | 20.5 | 0.2 |  | 30.2 | 27.3 | -2.8 |
| Jilin | 39.3 | 41.5 | 2.3 |  | 10.8 | 12.6 | 1.9 |
| Heilongjiang | 38.7 | 47.1 | 8.4 |  | 14.0 | 9.6 | -4.4 |
| Total | 30.6 | 29.8 | -0.9 |  | 15.3 | 19.0 | 3.7 |
| Note: Except that there was no statistically significant change in the low physical fitness of children in Beijing, Liaoning, Fujian and Shandong from 2005 to 2014, there were statistically significant differences in the two physical fitness levels in other provinces in two years. | | | | | | | |

| **Table S3.** The standardized values of each physical fitness components measurements, 2005 to 2014, China | | | | |
| --- | --- | --- | --- | --- |
| **Survey year** | **2005** | **2010** | **2014** | **2005-2014 Changes** |
| Forced Vital Capacity |  |  |  |  |
| East | -0.537 | -0.333 | -0.096 | 0.634 |
| Central | -0.822 | -0.682 | -0.314 | 1.136 |
| West | -1.253 | -0.985 | -0.797 | 2.051 |
| Northeast | -0.714 | -0.555 | -0.217 | 0.931 |
| Standing Long Jump |  |  |  |  |
| East | 0.442 | 0.533 | 0.355 | -0.797 |
| Central | 0.239 | 0.277 | 0.152 | -0.391 |
| West | 0.116 | 0.123 | -0.045 | -0.070 |
| Northeast | 0.074 | -0.023 | -0.110 | 0.036 |
| Sit and Reach |  |  |  |  |
| East | 0.141 | 0.253 | 0.229 | -0.371 |
| Central | 0.390 | 0.247 | 0.191 | -0.580 |
| West | 0.071 | 0.144 | 0.053 | -0.124 |
| Northeast | -0.027 | 0.018 | 0.011 | 0.016 |
| Body Muscle Strength |  |  |  |  |
| East | 0.558 | 0.557 | 0.486 | -1.044 |
| Central | 0.271 | 0.050 | 0.162 | -0.433 |
| West | 0.247 | 0.126 | 0.225 | -0.472 |
| Northeast | 0.625 | 0.275 | 0.404 | -1.029 |
| 50 Meter Dash |  |  |  |  |
| East | -0.276 | -0.333 | -0.323 | 0.600 |
| Central | 0.188 | 0.182 | 0.053 | -0.241 |
| West | 0.121 | 0.164 | 0.036 | -0.157 |
| Northeast | 0.001 | 0.247 | 0.139 | -0.140 |
| Endurance running |  |  |  |  |
| East | 0.843 | 0.786 | 0.818 | -1.662 |
| Central | 1.090 | 1.136 | 1.015 | -2.106 |
| West | 1.076 | 1.037 | 1.085 | -2.161 |
| Northeast | 1.041 | 1.623 | 1.492 | -2.533 |
